# Reintroduction of DJ-1 in Müller Cells Inhibits Retinal Degeneration in the DJ-1 Deficient Retina

**DOI:** 10.3390/antiox10121862

**Published:** 2021-11-23

**Authors:** Naouel Gharbi, Dagne Røise, Jorunn-Elise Førre, Amanda J. Edson, Helena A. Hushagen, Valentina Tronci, Ann-Kristin Frøyset, Kari E. Fladmark

**Affiliations:** 1 Integrative Fish Biology Group (IFB), NORCE Norwegian Research Center AS, N-5020 Bergen, Norway; nagh@norceresearch.no (N.G.); vatr@norceresearch.no (V.T.); 2 Department of Biological Science, University of Bergen, N-5020 Bergen, Norway; dagne.roise@uib.no (D.R.); Jorunn-Elise.Forre@student.uib.no (J.-E.F.); amandajedson@gmail.com (A.J.E.); helena.hushagen@student.uib.no (H.A.H.); ann.froyset@uib.no (A.-K.F.)

**Keywords:** DJ-1, oxidative stress, retina, Müller cell, neurodegeneration

## Abstract

The eye is continuously under oxidative stress due to high metabolic activity and reactive oxygen species generated by daily light exposure. The redox-sensitive protein DJ-1 has proven to be essential in order to protect retina and retinal pigment epithelium (RPE) from oxidative-stress-induced degeneration. Here, we analyzed the specific role of Müller cell DJ-1 in the adult zebrafish retina by re-establishing Müller-cell-specific DJ-1 expression in a DJ-1 knockout retina. Loss of DJ-1 resulted in an age-dependent retinal degeneration, including loss of cells in the ganglion cell layer, retinal thinning, photoreceptor disorganization and RPE cell dysfunction. The degenerative phenotype induced by the absence of DJ-1 was inhibited by solely expressing DJ-1 in Müller cells. The protective effect was dependent upon the cysteine-106 residue of DJ-1, which has been shown to be an oxidative sensor of DJ-1. In a label-free proteomics analysis of isolated retinas, we identified proteins differentially expressed after DJ-1 knockout, but with restored levels after Müller cell DJ-1 re-insertion. Our data show that Müller cell DJ-1 has a major role in protecting the retina from age-dependent oxidative stress.

## 1. Introduction

DJ-1 is a multifunctional and ubiquitously expressed protein encoded by the *park7* gene [1]. It is highly recognized as a general protector of oxidative stress through regulating antioxidant and anti-apoptotic gene transcription, as well as several distinct pro-survival pathways [2,3]. DJ-1 has a role in mitochondrial homeostasis and dynamics [4], in part through chaperone-mediated autophagy of damaged mitochondria [5], and was recently also shown to have a crucial role in mitochondria–ER interaction function [6].

DJ-1 function can be regulated through post-translational modifications of a highly conserved cysteine residue (Cys106) [7], which is recognized as an oxidative sensor. These modifications include both cysteine oxidation [7] and nitrosylation [8]. It should be noted, though, that some functions of DJ-1 also seem Cys106-independent [9,10].

The loss of DJ-1 causes an age-dependent increase in retinal abnormalities in DJ-1-deficient mice, including molecular and structural abnormalities, loss of photoreceptors, outer retina thinning and visual dysfunction [11,12]. DJ-1 seems to act similarly to an oxidative sensor in the retina; by inducing retinal oxidative stress, DJ-1 levels are upregulated [13].

Müller cells are the main glial cell in the vertebrate retina. They span the entire retina thus enabling them to interact with all retinal neurons. Müller cells are responsible for metabolic support of retinal neurons and obtaining retinal homeostasis [14]. Their neuroprotective function also comprises the release of antioxidants and neurotrophic factors [15]. Hence, understanding the basis of neuron–glial crosstalk is highly important. Müller cells are also of interest in regenerative therapies, as they are the stem cells of the retina [16]. 

Astroglial DJ-1 has proven to have a neuroprotective function in the brain by inhibiting oxidative-stress-induced death of dopaminergic neurons [17,18]. It was, therefore, of interest to explore whether Müller cell DJ-1 expression could have a similar role in the retina.

Zebrafish are highly valuable in vivo models due to their short regeneration time and accessibility for transgenesis. We have previously established a CRISPR/Cas9-based DJ-1-deficient zebrafish model [19]. Our first attempt was to elucidate if this model showed similar retinal degeneration as observed in rodents. By using glial fibrillary protein (*gfap*) promotor driven expression, it is possible to drive Müller-cell-specific expression in the retina [20,21]. Thus, by re-inserting DJ-1 under the control of the *gfap* promotor in the DJ-1 null background, we could study the role of Müller cell DJ-1 expression.

Loss-of-function mutation in DJ-1 causes an early onset form of familiar Parkinsonism [22]. Visual disorders are common in parkinsonism, but not specifically associated to any specific DJ-1 mutation [23]. Retinal thinning, however, has been suggested as an early biomarker of Parkinson’s disease as the more accessible eye can be a window to early pathologies [24]. The antioxidant function of DJ-1 is suggested to play a general role in several ocular neurodegenerative diseases [25]. Giving the accessibility and potential in transgenesis of zebrafish, modeling DJ-1 function in retina may be highly valuable in a translational perspective.

Here, we show that DJ-1 loss-induced structural retinal changes and changes in protein profiles can be inhibited by the retinal selective DJ-1 expression in the Müller cells and that this effect is dependent on the redox sensitive Cys106 residue.

## 2. Materials and Methods

Zebrafish were used to elucidate the function of Müller cell DJ-1 in retinal protection to oxidative stress. Transgenic lines with Müller specific expression of DJ-1 were established in a retinal DJ-1 null background. The function of Müller DJ-1 was studied by using a combination of morphological analysis and protein profiling by mass spectrometry.

### 2.1. Animal Maintenance

Animals were housed in the zebrafish facility located in the Department of Biological Science at the University of Bergen. The facility is run according to the European Convention for the Protection of Vertebrate Animals used for Experimental and Other Scientific Purposes. Adult zebrafish were maintained at 26–28 °C, with a 14/10 light cycle and were fed twice daily.

Embryos were maintained at 28 °C and raised in E3 buffer (5 mM NaCl, 0.17 mM KCl and 0.33 mM MgSO_4_) until 12 days post-fertilization (dpf).

### 2.2. Zebrafish Lines

We have previously published the *dj1*^–/–^ (KO) line [19]. This line was established by using the CRISPR-Cas9 method, targeting the exon 1 of *park7*, and approved by the National Animal Research Authority at Mattilsynet (FOTS ID8039 and ID14039). Zebrafish with selective DJ-1 expression in glia, *dj1*^−/−^; Tg(*gfap:eGFP-2a-dj1*), were established by insertion of pBs-ISceI-gfap:eGFP-2A-Flag-DJ-1 [17] into the *dj*^–/–^ line. QuickChangeII Site-Directed Mutagenesis Kit (Agilent, Technologies, Santa Clara, CA, USA) was used to perform mutagenesis on pBs-ISceI-gfap:eGFP-2A-Flag-DJ-1 to introduce the C106A mutation in DJ-1.

Transgenesis was performed by injection of 0.5 nL restriction digest: plasmid (0.6 μg), injection dye (0.5% phenol red, 240 mM KCl and 40 mM HEPES, pH 7.4) 1 μL, 10Å ~ I-Sce1 buffer 0.5 μL, I-SceI (New England BioLabs: R0694S, Ipswich, MA, USA) 1 μL, ddH2O to total 10 μL, into single-cell embryos. Embryos expressing eGFP were selected 48 hpf and raised to adulthood.

Founder fish were outcrossed with wild-type and progeny embryos (F1) collected. Stable lines were expanded from single F1 founders, and eGFP expression in transgenic animals was examined by using a fluorescent Zeiss SteREO Lumar microscope.

### 2.3. Eye Sectioning, Toluidine Blue Staining and Image Analysis

The eyes of adult zebrafish (3–18 months) were fixed in 4% paraformaldehyde for 48 h at 4° and were washed and rehydrate in (50%, 70% and 96%) EtOH. Eyes were pre-infiltrated for 4 h, at room temperature, in 50%(96%) EtOH/50% base liquid (Kulzer Technovit^®^ 7100: 64-7090-03, Kulzer GmbH, Wehrheim, Germany). They were then infiltrated in preparation solution overnight at room temperature (see user instruction). Eyes were oriented in the polymerization solution at room temperature and left overnight. Then 2 μm sections were prepared by using a Leica Microtome (RM 2165, Leica Biosystems, Nussloch, Germany). Sections were dried before and after staining with 1% toluidine blue. The sections were mounted in DPX (Sigma:06522, Merck, Damstadt, Germany). Images were obtained by using Zeiss Axio Scan.Z1.

All image processing and measurements were performed in ImageJ2 version 2.1.0/1.53c. Selections containing the retina were created for each image, and binary images were created from these selections. Retinal thickness was then measured by using the “Average thickness” plugin (part of MorphoLibJ [26]). The number of cells in the ganglion cell layer was counted manually from sagittal eye sections, including *n.opticus*. Statistical analysis was performed in Prism 9.2.0 for macOS (GraphPad Software, San Diego, US) using one-way ANOVA and Tukey’s multiple comparisons test.

### 2.4. Cryo-Sectioning

Zebrafish eyes were fixed in 4% paraformaldehyde for 24 h, at 4 °C, and cryoprotected overnight in 25% (OCT) compound medium in 25% sucrose solution. Eyes were oriented in a plastic mold in 100% OCT, cooled down and stored at -80 °C for sectioning [27]. Then 10 μm serial sections were prepared at −25 °C, using a Leica CM1800 cryostat.

### 2.5. In Situ Hybridization

Plasmids containing gstp1 (cb356) cDNA fragments were purchased from ZIRC (http://zebrafish.org/zirc/home/guide.php, 10/September/2020). Sense and antisense RNA probes were synthesized from the amplified template, using the recommended polymerases and Digoxigenin-labeled ribonucleotides (Roche: Cat. No. 11093274910). In situ hybridization for gstp1 transcript (sense and antisense) was performed on 10 μm serial sections (*n* = 6) of adult zebrafish eyes. Tissue pretreatment and in situ hybridization steps were performed as described previously [28].

### 2.6. Protein Extraction

Resected retinas from 9-month-old zebrafish were homogenized in 150 µL homogenization buffer (10 mM K_2_HPO_4_, 10 mM KH_2_PO_4_, 1 mM EDTA, 0.6% CHAPS, 0.2M Na_3_VO_4_, 50 mM NaF, protease cocktail (Roche, Mannheim, Germany)) and sonicated 4 × 5 s on ice. Samples were pelleted by centrifugated at 13,000 *g* for 15 min. The protein extract was kept frozen at −80 °C until further processing.

### 2.7. Verification of Transgenic Line

Brain tissue from eye resected animals were homogenized, as explained above for retinas. Proteins were separated on Invitrogen™ NuPAGE™ 4–12% Bis-Tris gels and blotted onto PVDF membranes. Membranes were stained with Ponceau S before blocking in 1% BSA/PBS-T. Membranes were further incubated with anti-DJ-1 (Novus NB300-270, 1:3000, Novus Biologicals, Abingdon, UK), followed by anti-rabbit, and developed by using ECL substrate (ThermoFisher scientific, Sunnyvale, California, CA, USA). The eGFP expression was verified by fluorescent microscopy on frozen sections of retinas, using anti-GFP (Invitrogen A6455 1:2000, ThermoFisher, Sunnyvale, California, CA, USA) and anti-rabbitAF647 (Invitrogen A21245, 1:500, ThermoFisher, Sunnyvale, California, CA, USA). Labeling with a far-red fluorescent dye was chosen to avoid interference with autofluorescence from photoreceptors.

### 2.8. Transmission Electron Microscopy

Resected eyes were washed in 0.1 M sodium cacodylate buffer and fixed in 1.5% glutaraldehyde in 0.1 M Na-cacodylate buffer, pH 7.4, overnight. Eyes were then washed in 0.1 M sodium cacodylate buffer and post-fixed in 1% osmium tetroxide in 0.1 M sodium cacodylate buffer for 60 min. Eyes were washed twice for 15 min in 0.1 M sodium cacodylate buffer and dehydrated in graded solutions of ethanol. Eyes were then embedded in plastic, using Agar 100 resin, cut into 60 nm thin sections (Reichert Ultracut S Ultra microtome, Leica Biosystems, Nussloch, Germany) and stained in uranyl/lead. Sections were examined by using a Jeol JEM-1230 transmission electron microscope at the UiB Core Facility for Imaging.

### 2.9. Isolation of Retina for Mass Spectrometry

Retinas were sampled from age-matched animals (9 months) between 9:00 and 10:00 a.m. Animals were first euthanized with MS-222 in facility water and thereafter transferred to an ice bath. The cornea was cut, and the lens was taken out in situ under a stereo microscope. The eye was thereafter lifted out, and the *n.opticus* was cut. The retina was then collected by pushing back the sclera, using two forceps, and immediately frozen in liquid nitrogen.

### 2.10. Sample Preparation for Mass Spectrometry

In total, 30 µg of protein extract was denaturated with 200 µL 8M urea on Microcon YM-30 (#MRCF0R030, Merck KGaA, Darmstadt, Germany) according to Wisniewski et al., in 2009 (FASP protocol), followed by reduction, alkylation and trypsin digestion as described in Frøyset et al. (2016) [29]

### 2.11. Label-Free Mass Spectrometry

Tryptic peptides 0.5 µg were analyzed on Ultimate 3000 RSLC system (Thermo Scientifi, Sunnyvale, California, CA, USA) connected online to a QExactive HF mass spectrometer (Thermo Scientific, Bremen, Germany). The sample was loaded and desalted as previously described in Frøyset et al. [29].

The peptides were separated during a biphasic ACN gradient from two nanoflow UPLC pumps with flow rate 250 nL/min on a 25 cm analytical column (PepMap RSLC, 25 cm × 75 µm ID EASY-spray column, packed with 2 µm C18 beads, Thermo scientific, Waltham, MA, USA). Solvent A and B were 0.1% FA (vol/vol) in water and 100% ACN, respectively. The gradient composition was 5% B during trapping (5 min), followed by 5–7% for 0.5 min, 7–22% B for the next 44.5 min, 22–35% over 15 min and 35–80% B over 5 min. Elution of very hydrophobic peptides and conditioning of the column were performed during a 7-min isocratic elution with 80% B and 10-min isocratic conditioning with 5% B. The total length of the LC run was 90 min.

MS spectra were acquired as described in Reference [29], but with minor/some changes. The instrument control was through Q Exactive HF Tune 2.9 and Xcalibur 4.1. MS spectra were acquired in the scan range 375–1500 m/z with resolution R = 120,000 at m/z 200, automatic gain control (AGC) target of 3e6 and a maximum injection time (IT) of 100 ms. The 12 most intense eluting peptides above intensity threshold 40,000 counts, and charge states 2–5 were sequentially isolated to a target value (AGC) of 1e5 and a maximum IT of 118 ms in the C-trap, and isolation with maintained at 1.2 m/z (offset of 0.3 m/z), before fragmentation in the HCD (Higher-Energy Collision Dissociation) cell. Fragmentation was performed with a normalized collision energy (NCE) of 28%, and fragments were detected in the Orbitrap at a resolution of 60,000 at m/z 200, with first mass fixed at m/z 120. One MS/MS spectrum of a precursor mass was allowed before dynamic exclusion for 20 s with “exclude isotopes” on. Lock-mass internal calibration (m/z 445.12003) was used. The ion source parameters were as follows. Ion spray voltage = 1800 V, no sheath and auxiliary gas flow; and capillary temperature was 275 °C.

### 2.12. Data Interpretation

The raw files were searched in MaxQuant (version 1.6.0.16) against UniProtKB (reviewed and un-reviewed) database with 62016 entries (downloaded 28 January 2020). The same settings as described in Frøyset et al. [29] were used in MaxQuant search and Perseus (version 1.6.6.0) in further analysis. The different lines were compared to WT (*n* = 3): *dj1*^–/–^, *dj1*^–/–^*;Tg(gfap:eGFP-2A-dj1)*, and *dj1*^–/–^*;Tg (gfap:eGFP-2A-dj1_c106a_)*. The protein list was further reduced by only accepting proteins with minimum 3 valid values in at least one group. The LFQ intensity values were log2 transformed, and the proteins were considered significant if they passed the two sample t-test with the following settings; S0 = 2, and *p*-value 0.05.

## 3. Results

### 3.1. Generation of Transgenic Zebrafish Lines with Müller Cell Specific Wild Type DJ-1 and DJ-1c106a Expression in a DJ-1 Null Background

**Figure 1:**
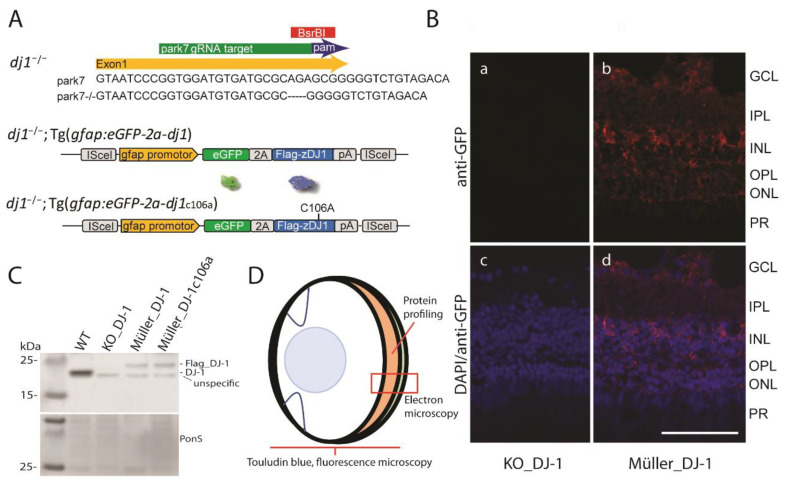
Zebrafish lines and workflow. (**A**) A DJ-1 knockout line was previously established by using the CRISPR-cas9 method to target a 20 bp region of exon one of the park7 gene [19]. Lines expressing glial specific wild-type DJ-1 or DJ-1 c106a in a DJ-1 null background were constructed by using ISce1 transgenesis and regulatory elements of glial fibrillary acidic protein (GFAP). The viral 2A peptide allows expression of GFP and Flag-DJ1 as uncoupled protein. In the retina, the gfap promotor drives expression only in the Müller glia cells. (**B**) Glial expression of GFP in the Müller_DJ-1 line (**b**,**d**) versus KO_DJ-1 (**a**,**c**). Note that GFP expression was determined by using a GFP antibody, followed by a far-red secondary antibody to avoid interference from retinal autofluorescence. Prominent expression is found around the Müller cell bodies. GFP expression also extends through the Müller processes into the photoreceptor layer. Faint expression can be observed in the Müller foot processes on the inner limiting membrane. Bar, 50 μm. (**C**) A Western blot shows expression of endogenous DJ-1 and Flag-tagged DJ-1 from total brain extracts belonging to animals from which eyes were collected. Ponceau S staining was used as a loading control. Asterisk points to an unspecific band. (**D**) Sagittal view of the zebrafish eye and workflow employed in this study.

We have previously established a DJ-1-deficient zebrafish line [19]. This line was generated by using the CRISPR-Cas9 method to target exon 1 of the *park7* gene to knockout DJ-1 (Figure 1A). Here, we have reinserted DJ-1 and DJ-1_c106a_ in glia cells of the knockout line, using ISce1-transgenesis and elements of the glia fibrillary acidic protein (*gfap*) promotor to enable glial specific expression of DJ-1. In the retina, however, this glial expression is restricted to the Müller cells [20,30], thus making it possible to study the effect of Müller specific DJ-1 expression in a retinal DJ-1 null background. Flag-tagged DJ-1 and mutant are expressed together with GFP, but separated by the viral 2A peptide, which allows stoichiometric unfused expression of the proteins (Figure 1A–C). Moreover, eGFP expression was prominent around the Müller cell bodies and could also be observed in their processes extending to the photoreceptor layer (Figure 1B). Less GFP expression was observed extending towards the inner limiting membrane. Eyes from the three zebrafish lines, namely *dj1*^–/–^ (DJ-1_KO), *dj1*^–/–^*;Tg(gfap:eGFP-2A-dj1)* (Müller_DJ-1), and *dj1*^–/–^*;Tg(gfap:eGFP-2A-dj1_c106a_)* (Müller_DJ-1_c106a_), together with wild-type eyes, were used in this study to evaluate the role of Müller cell expressed DJ-1 in retinal neuronal protection from oxidative stress induced by the loss of DJ-1 (Figure 1D). Mass-spectrometry-based analysis of isolated retinas showed that Müller cells expressed DJ-1 and mutant DJ-1 levels were 0.10 and 0.18 fold, respectively, when compared to endogenous DJ-1 levels in wild-type whole retina (Supplementary Materials Table S2).

### 3.2. Retinal Degeneration Induced by the Loss of DJ-1 Can Be Inhibited by Müller Cell Expressed DJ-1

**Figure 2:**
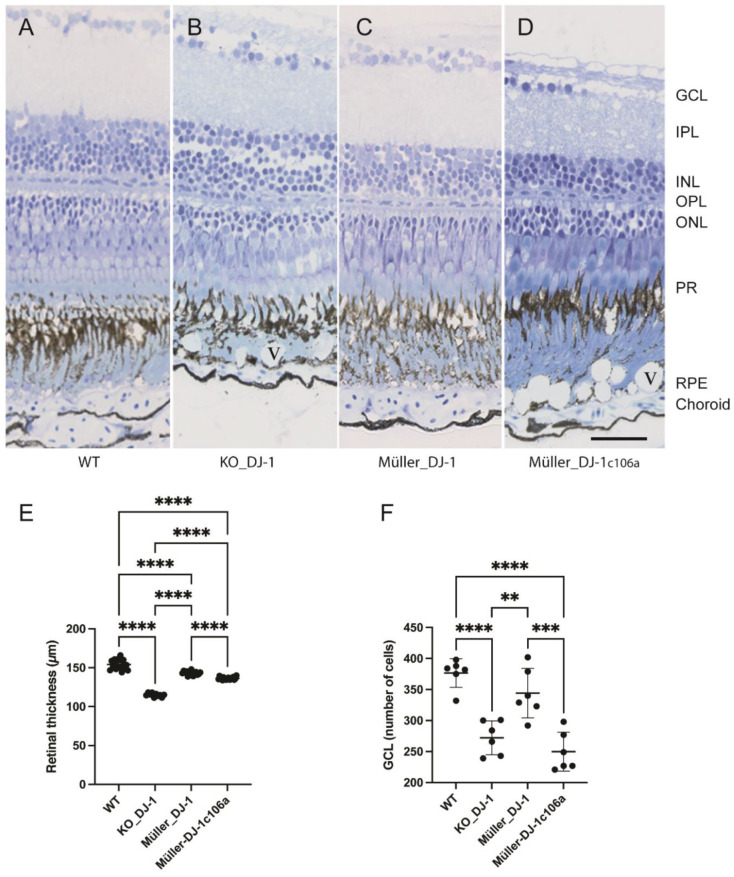
Laminar morphology of wild-type, knockout and transgenic retinas. Light microscopic images of toluidine-blue-stained retinal cross-sections from nine-month-old adult zebrafish: (**A**) wild type, (**B**) DJ-1 knockout, (**C**) Müller-cell-expressed wild-type DJ-1, (**D**) Müller-cell-expressed DJ-1c106a mutant. (**E**). Retinal thickness (μm) measured on eye sections from three fish in each group. **** *p* < 0.0001, one-way ANOVA, *n* = 17 (WT), 10 (KO_DJ-1), 15 (Müller_DJ-1) and 19 (Müller_DJ-1c106a). (**F**) Number of cells in ganglion cell layer measured on sections from three fish; ** *p* < 0.01, *** *p* < 0.001 and **** *p* < 0.0001 versus wild type, one-way ANOVA, *n* = 6). GCL, ganglion cell layer; IPL, inner plexiform layer; INL, inner nuclear layer; OPL, outer nuclear layer; PR, photoreceptors; RPE, retinal pigment epithelium; V, vacuole in RPE layer. Bar, 20 μm applies to all panels.

An earlier study of retina from DJ-1 knockout mice showed that loss of DJ-1 mainly affected the outer plexiform layer, photoreceptors and retinal pigment epithelium (RPE) [12,31]. Toluidine-blue-stained semithin sections of retina from 1-to-18-month-old DJ-1_KO zebrafish showed an age-dependent degeneration of retina (Supplementary Figure S1. In 5-month-old animals, the loss of DJ-1 expression seemed to have little effect on the structure and morphology of the retina, but in 9-month-old animals a marked change could be observed with vacuole-like structures in the retinal pigment epithelial (RPE) layer and disruption of photoreceptor filament organization (Figure 2). These changes seemed to be even more manifested in older animals (Supplementary Figure S1).

We focused on 9-month-old retinas and included the analysis of the transgenic animals with reintroduced Müller-specific wild-type and mutant DJ-1 expression in the DJ-1 null background (Figure 2). Morphological changes observed in the DJ-1_KO included both a retinal thinning and a reduction of cells in the ganglion cell layer, in addition to the morphological changes observed in the RPE and photoreceptor layers (Figure 2). This retinal degenerative pathology was inhibited by selective Müller cell DJ-1 expression. Reintroducing Müller cell DJ-1_c106a_, on the other hand, did only inhibit changes in the photoreceptor layer and to some degree retinal thinning. Thus, distinct Müller cell DJ-1-dependent pathways seem to be involved in retinal protection, with at least one being C106-dependent.

### 3.3. Introduction of Müller Cell DJ-1 Expression Inhibits DJ-1 Loss-Induced Ultrastructural Changes in the Retinal Pigment Epithelia Cells

**Figure 3:**
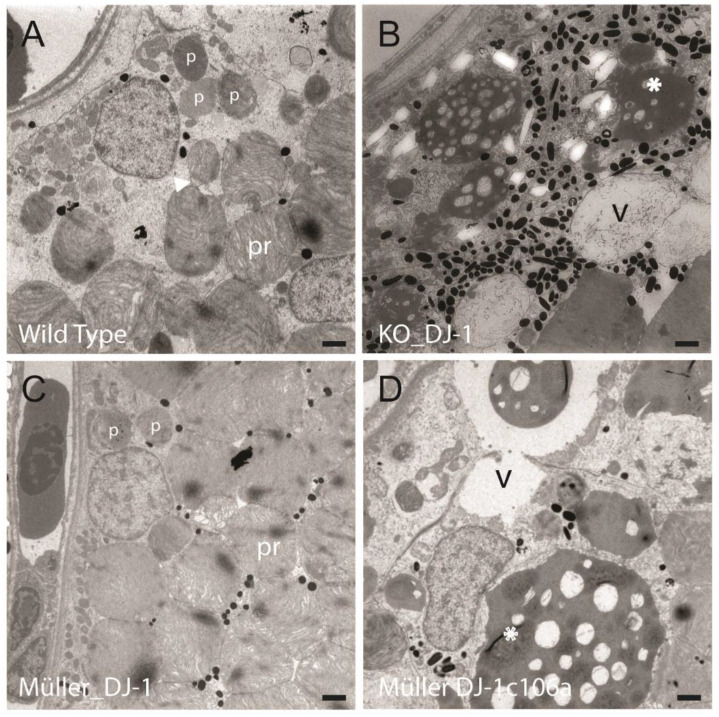
Ultrastructural changes in the retinal pigment epithelium induced by the loss of DJ-1 are inhibited by introducing DJ-1 in Müller cells. Electron micrographs of photoreceptors (pr) and retinal pigment epithelium (RPE) of wild-type (**A**), DJ-1 knockout (**B**), Müller-expressed DJ-1 (**C**) and Müller-expressed DJ-1c106a mutant (**D**) retinas from 4-month-old adult zebrafish. DJ-1-deficient retinas displayed a high degree of vacuolization (v), in addition to large electron-dense structures (*) in the RPE. The large electron-dense structures contained remnants of membranous/filamentous structures. Transgenic retinas expressing Müller-selective DJ-1c106a mutant displayed similar morphology as DJ-1 knockouts. The ultrastructural changes induced by DJ-1 loss were prevented by Müller cell selective expression of wild type DJ-1. Different stages of phagosomes containing segment filaments can be seen in wild-type and Müller_DJ-1 retinas (marked with arrowhead and *p*). Bar, 1 µm.

**Figure 4:**
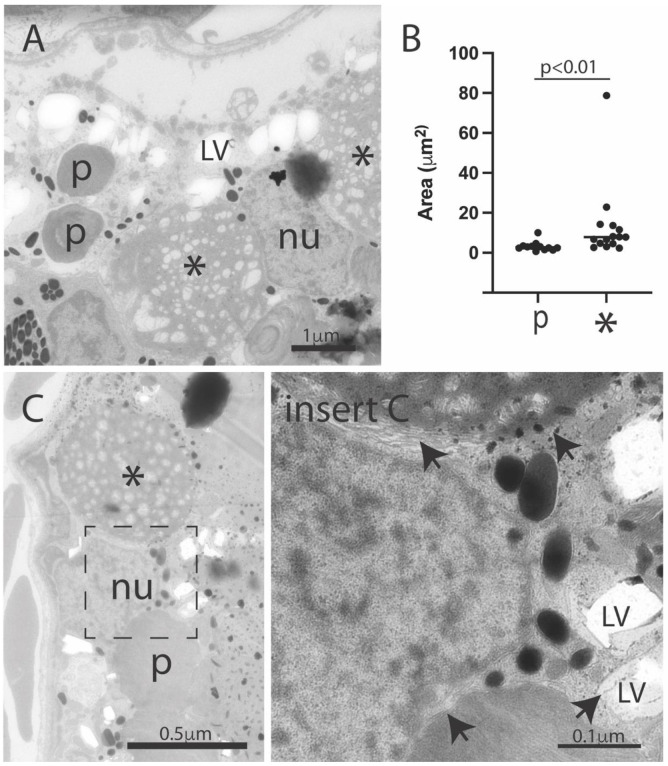
Ultrastructural features in the retinal pigment epithelium of the DJ-1-deficient retina. Electron micrographs of the RPE area of DJ-1 knockouts (**A**,**C**). Images show both phagosomes (*p*) and the larger electron-dense structures (*), in addition to numerous small electron lucent vacuoles (LV) in RPE. Phagosomes and electron lucent vacuoles were surrounded by membranes (arrows), but no clear limited membrane was observed limiting the electron-dense structures. (**B**) Quantitation of area of phagosomes (*p*) and electron-dense structures (*); *p* < 0.01. Mann-Whitney-test, n = 12 (WT) and 15 (KO_DJ-1); *p*, phagosome; * electron-dense structure; LV, electron-lucent vacuole; nu, RPE nucleus.

RPE cells have an important role in diurnal phagocytosis of photoreceptors, as distal ends of their outer segments are pinched off and phagocytosed by neighboring RPE cells before they are degraded in a lysosomal-dependent pathway [31]. Different stages of phagosomes could be observed in all samples (Figure 3 and Figure 4, marked arrowhead and *p*), but in contrast to wild type and Müller_DJ-1, DJ-1_KO and Müller_DJ-1c106a contained a number of large electron-dense structures (Figure 3 and Figure 4, marked *). The morphometric analysis of these structures in the DJ-1_KO showed a mean cut diameter of 3.9 μm; thus, they are far larger than the phagosomes (0.9 μm) (Figure 4B). Their electron-density and filamentous content resembled the appearance of heterolysosomes, but somehow they seemed to have been arrested in their degradation process. In the RPE cells of DJ-1_KO and Müller_DJ-1c106a, these large electron-dense structures would occupy most of the cytoplasmic space. In both DJ_KO and Müller_DJ-1c106a, large vacuoles in the RPE area were present. These vacuoles most possibly resemble the vacuoles observed at the light microscopic level (Figure 2).

A number of electron-lucent vacuoles were observed in the DJ-1 knockout RPE (Figure 4, marked LV). These structures may possibly be lipid vesicles in which lipid content is partly washed out during tissue preparation [32].

### 3.4. Protein Profiling of Resected Retinas

In an attempt to identify proteins associated with the loss of DJ-1 and proteins associated with Müller DJ-1 protection, we performed a label-free quantitative global mass-spectrometry-based protein analysis. NanoLC-HDMSE analysis was carried out on resected retinas from three animals from each of the groups, namely wild type, DJ-1_KO, Müller_DJ-1 and Müller_DJ-1_c106a_, at the age of nine months. A total of 3211 proteins were identified on the basis of one or more peptides with a mass accuracy ≤ 10 ppm and a score ≥ 4. For further quantitative analysis, the protein should be identified with at least two unique peptides and appear in all three samples in at least one group, leaving 1868 proteins. It should be noted that, because the zebrafish proteome has not yet been comprehensively annotated with gene ontology (GO) terms, our data interpretation, in some cases, relies on the knowledge of their mammalian orthologue.

All protein hits qualifying for quantitative analysis are listed in Supplementary Materials Table S2.

### 3.5. Expression of Retinal Cell Markers in Knockout and Transgenic Lines

To establish the level of possible retinal degeneration and/or gliosis after DJ-1 loss, we searched for cell specific markers of Müller cells, retinal epithelial cells (RPE), rod photoreceptors, cone photoreceptors, retinal ganglion cells and microglia/macrophages [20,33,34] (Supplementary Materials Table S1).

No sign of gliosis, as reflected by an increase in Müller cells markers (GFAP and Glutamate synthase), was observed. On the other hand, the ganglion marker Gefiltin and a Rhodopsin variant associated to rod cells were decreased in knockout compared to wild-type retina.

The significant decrease in rhodopsin variant was also observed in Müller_DJ-1c106a.

### 3.6. Loss of DJ-1 Alters Expression of Proteins Belonging to the Respiratory Complex I and Glycolysis Independently of Reinsertion of Müller Cell DJ-1

To get an overview of proteins regulated by loss of DJ-1, we selected proteins with expression levels altered in DJ-1_KO, even though wild-type or mutant DJ-1 were re-introduced in the retinal Müller cells (Table 1). Most probably these identifications reflect protein changes in the neuronal retina or RPE. A majority of these proteins were components of the mitochondrial complex I. All of them were significantly downregulated in DJ-1_KO, Müller_DJ-1 and Müller_DJ-1_c106a_, as compared to wild-type retinas. On the contrary, lactate hydrogenase, which converts pyruvate to lactate in glycolysis, was upregulated. Possibly, these changes reflect a shift in metabolism to minimize oxidative stress [35]. Another seeming response to oxidative stress was the upregulation of both glutathione S-transferase and glutathione peroxidase in both knockout and transgenic retinas compared to wild type. A corresponding transcriptional upregulation of Glutathione S-transferase was verified by using in situ hybridization (Supplementary Materials Figure S2). The in situ hybridization showed, in particular, high transcriptional levels of Glutathione S-transferase in the ganglion cell layer and inner nuclear layer in both knockout and Müller_DJ-1c106a as compared to wild type and Müller_DJ-1.

### 3.7. Identification of Retinal Proteins Regulated by the Loss of DJ-1, but with Restored Levels after Introducing Müller Cell DJ-1

The morphological analysis showed that structural changes and retinal degeneration in DJ-KO were inhibited by the introduction of wild-type DJ-1 in Müller cells, but not by its mutant form. We therefore searched for retinal proteins dysregulated in both DJ-1_KO and Müller DJ-1_c106_, but with wild-type expression levels in Müller_DJ-1 (Table 2). Within these criteria, we identified Prosaposin and G-protein-coupled receptor 37a (Gpr37), which are involved in glial-neuron protection [36]. Additionally, we also identified proteins regulating cell structure (Calponin 2), mitochondrial motility (Metaxin), autophagy (SEC23-interacting protein) and inflammation (serum Amyloid P component) to be differentially expressed [37,38,39]. Expression levels for a ribosomal protein (Rpl36a) and a nuclear export protein (Exportin 1) were also found to be altered.

### 3.8. Identification of Proteins with Altered Expression in DJ-1 Knockouts Regardless of Introducing Either Müller Cell DJ-1 or Müller Cell DJ-1c106a

Although the cysteine-106 residue of DJ-1 is considered as an oxidative sensor through cysteine oxidation [7], DJ-1-dependent antioxidant function has also proven to be independent of C106 [10,40]. Introducing mutant DJ-1 in Müller cells did not protect from the retinal degeneration induced by DJ-1 loss (Figure 2, Figure 3 and Figure 4). Intriguingly, it seemed to prevent a general stress response, a response that might be neuroprotective (Table 3). Seven proteins were found to be upregulated only in the DJ-1 knockout: Ependymin, Histone-H1-like, Cathepsin D, Methylmalonyl coA epimerase, Crystallins and Grifin. Crystallin gamma 1 and 2a, and Grifin, known as lens proteins, have all previously been found to be upregulated in retina as a response to stress [41]. Increasing evidence shows that Crystallins may have an important antioxidant function besides being structural lens proteins [42,43]. Methylmalonyl CoA epimerase is involved in lipid catabolism. Cathepsin D is an essential lysosomal protease in RPE cells and Cathepsin D deficiency results in extensive accumulation of lipofuscin [44]. Ependymin, an extracellular lipid-binding protein, is involved in cell adhesion and neuronal regeneration [45].

### 3.9. Discussion

In the present study, we show that loss of DJ-1 in zebrafish induces an age-related retinal degeneration similar to what has been observed in DJ-1-deficient mice [46] and to retinal pathologies associated with neurodegenerative diseases [47]. Here, however, we also demonstrate that this DJ-1 loss-induced degenerative retinal phenotype can be inhibited by reintroducing DJ-1 selectively in the retinal Müller cells.

Müller-specific DJ-1 expression was enabled by expressing DJ-1 under control of elements of the *gfap* promotor into a DJ-1 knockout line [19]. This promotor drives expression in astrocytes in the brain, but in retina, it is a Müller-specific promotor and does not drive expression in neither retinal astrocytes or microglia (Figure 1A,B) [20,30]. Both wild-type DJ-1 and DJ-1_c106a_ were expressed, the latter because the Cysteine 106 is believed to act as an oxidative sensor in DJ-1 and to be essential for at least parts of DJ-1’s antioxidant response pathways [10,40].

DJ-1 is a multifunctional protein with its importance in oxidative-stress protection being the most recognized. DJ-1 is expressed in all cell types, but in the brain, astrocytic expression seems highly important, as astrocyte DJ-1 not only protects the astrocytes themselves from oxidative stress, but also neighboring neurons [17,18]. Moreover, elevated DJ-1 expression within activated astrocytes is a pathological feature found in several neurodegenerating diseases, including Parkinson’s disease and Alzheimer’s disease [48,49]. Müller cells, the predominant glia cells in the retina, exhibit a comparable function in the retina as to astrocytes in the brain [50].

The loss of DJ-1 induced an age-dependent degeneration of the ganglion cell layer, prominent morphological changes in the retinal pigment epithelial cells (RPE) and structural changes in the photoreceptor layer (Figure 2, Figure 3 and Figure 4 and Supplementary Materials Figure S1). The morphological changes in the RPE layer included vesiculation and occurrence of large electron dense structures, with the latter almost occupying the entire cytosol of the RPE cells in the aging DJ-1-deficient retina. All degenerative morphological features were inhibited by reintroducing Müller-selective wild-type DJ-1, but not DJ-1_c106a_ (Figure 2, Figure 3 and Figure 4).

No sign of gliosis, as reflected in increased expression of Müller cells markers, was observed in DJ-1-deficient or transgenic retinas (Supplementary Materials Table S1). On the other hand, both DJ-1 knockout retinas and retinas with Müller-specific DJ-1c106a expression showed increased levels of the inflammatory markers Serum Amyloid P component and Prosaposin (Table 2). Both have been associated with Parkinson’s disease [51,52].

Our proteomic profiles of whole retinas showed that the loss of DJ-1 affected the central metabolism by downregulation proteins belonging to the mitochondrial complex I and upregulating lactase hydrogenase, which converts pyruvate to lactate (Table 1). This most probably reflects a metabolic shift from oxidative phosphorylation to glycolysis to lower production to reactive oxygen species [53,54]. A similar change in protein profile was also observed in the lines with Müller cell DJ-1 expression, but to a lower degree. A metabolic shift would primarily affect the retinal neurons, and in particular the ganglion cells, which heavily depend on mitochondrial metabolism in contrast to Müller cells [55]. Both DJ-1 knockout and Müller DJ-1-expressing lines showed an upregulation of the oxidative-stress-response proteins glutathione peroxidase and glutathione S-transferase (Table 1), but seemingly this response was not sufficient in order to protect from retinal degeneration. Interestingly, DJ-1 knockout retinas also showed an upregulation of other proteins associated with retinal oxidative-stress response and with antioxidant properties [41,42,43,56,57]: the lens proteins Crystallins and Grifin (Table 3). This upregulation, which might have neuroprotective properties, was not observed in either retina expressing Müller DJ-1 wild type or mutant, thus indicating that both wild-type and mutant form DJ-1 in Müller cells induce an antioxidative response, which avoids initiating this alternative stress response.

Müller cells have an important function in retinal redox homeostasis, as they release glutathione (GSH), the major retinal antioxidant [58]. The tripeptide GSH is synthesized from serine/cysteine, glycine and glutamate, in which the latter is released from surrounding neurons and taken up into Müller cells via EAAT transporters [58]. DJ-1 may have several ways to influence and maintain GSH metabolism in Müller cells. Both glutamine influx and serine metabolism, which provide precursors of GSH synthesis, are reduced in DJ-1-deficient cells [59]. De novo synthesis of serine has shown to be important for Müller cell resistance to oxidative stress [60]. DJ-1 can also increase the uptake of neuronal released glutamate [61], which, in addition to reducing excitotoxicity, indirectly stimulates the Nrf2 pathway [62]. DJ-1 may also influence the availability of GSH by regulating rate-limiting enzymes in GSH synthesis [63]. Thus, reintroducing DJ-1 in Müller cells would not only re-establish redox balance in the Müller cell itself, but also the oxidative-stress-response pathway by which the surrounding DJ-1-deficient retinal neurons and RPE cells depend on.

Müller cells also have an important function in structural organization and assembly of photoreceptor outer segments (POSs), and targeted disruption of Müller cell metabolism affects the assembly of POS [64]. In the DJ-1 knockout retina, POSs appear to be unstructured, whilst both retinas expressing wild-type and C106-mutant DJ-1 in Müller cells appear to maintain proper POS organization (Figure 2).

Our proteomics analysis also suggested a possible role of Müller cell DJ-1 in regulating the neuroprotective prosaposin/GRP37 pathway (Table 2). Prosaposin (PSAP) is a neurotrophic factor mediating its neuroprotective effect through astrocytic GRP37L1 and GRP37 receptors [36]. In both DJ-1 knockout and Müller DJ-1_C106A_-expressing retinas prosaposin levels were increased, whereas GRP37a, an ortholog to human GPR37, was only observed in wild-type and Müller DJ-1 retinas (Table 2). Transcriptional profiling and in situ hybridization of mouse retina have shown that Müller cells are enriched in GRP37 transcripts [65]. Müller DJ-1 may potentially regulate Prosaposin/GPR37 signaling both through its regulation of the C106-dependenten ERK1/2 signaling [66] and through its regulation of PARKIN, which has GPR37 as a substrate [8,67].

Both DJ-1 knockout and retinas only expressing DJ-1_C106A_ in Müller cells showed age-dependent changes in the RPE cell layer, with accumulation of vesicles and electron dense structures (Figure 2, Figure 3 and Figure 4). RPE cells phagocytose and digest daily shed photoreceptor outer segments (POSs) though a lysosomal-dependent pathway [31]. We observed different stages of phagosomes in the RPE of all zebrafish lines, but the much larger electron-dense structures were only observed in the knockout and Müller mutant DJ-1-expressing line (Figure 3 and Figure 4). We are unsure of the identity of these structures, but they seemed to include POS-like structures. Thus, indicating that both DJ-1-deficient retinas and Müller DJ-1c106a-expressing retinas, in contrast to Müller wild-type DJ-1-expressing retinas, are dysfunctional in their degradation process of POS. RPE cells in both knockout and Müller cell DJ-1c106a-expressing retinas may be subjected to higher oxidative stress levels and non-degradable components in POS, thus hampering their normal function in POS phagocytosis and degradation [68]. The increase of the lysosomal Cathepsin D and lipid metabolizer Methylmalonyl CoA epimerase in knockout retinas possibly reflects high lysosomal stress in RPE cells (Table 3). Calponin, which plays a role in cell migration and phagocytosis, showed altered expression levels in DJ-1 knockout and Müller cell DJ-1c106a-expressing retinas, as compared to wild-type and Müller DJ-1-expressing retinas (Table 2). It should be noted that zebrafish and also other vertebrate Müller cells are able to phagocytose cell debris from degenerating photoreceptors [69]. This function may be dysregulated in Müller cell DJ-1-deficient cells as DJ-1 has been proposed to be an activator of phagocytosis [70].

In conclusion, we have shown that loss of retinal DJ-1 induces an inflammatory and antioxidative response. This stress response is not sufficient to avoid severe age-dependent retinal degradation. In contrast, through re-insertion of DJ-1 selectively in Müller cells, the retinal degradation is avoided. This rescue effect is dependent upon the oxidative-stress sensor C106 residue of DJ-1. The Müller cell DJ-1 function seems to involve both regulation of retinal redox homeostasis and possibly also the psap/GPR37 neuroprotective pathway.

## Figures and Tables

**Table 1:** Proteins regulated in the DJ-1-deficient, Müller DJ-1-expressing, and Müller DJ-1c106a-expressing retinas compared to wild type.

						*p*-Values (vs. WT)			Max Fold			Average LFQ (log 2)			
Protein ID	Gene Name	Protein Name	Total Peptides	Unique Peptides	Score	KO	M_DJ-1	M_DJ-1c106a	KO/WT	M_DJ-1/WT	M_DJ-1c106a/WT	Wild Type	KO	M_DJ-1	M_DJ-1c106a
**Mitochondrial Complex I**															
E9QEE8	ndufb4		3	3	31	***	**	***	0.3	0.4	0.5	25.38 ± 0.08	23.43 ± 0.29	23.89 ± 0.30	24.35 ± 0.15
Q498W6	ndufa12		4	4	38	*	**	*	0.3	0.4	0.5	26.04 ± 0.20	24.41 ± 0.51	24.79 ± 0.13	25.01 ± 0.4
Q6P6E5	ndufb7		4	4	60	*	**	*	0.3	0.6	0.7	26.44 ± 0.10	24.89 ± 0.54	25.59 ± 0.18	25.83 ± 0.18
Q6DGM9	ndufa7		3	3	22	**	**	*	0.4	0.5	0.5	25.61 ± 0.09	24.14 ± 0.43	24.49 ± 0.20	24.66 ± 0.47
Q6PBJ6	ndufb6		3	3	23	***	*	*	0.4	0.4	0.5	25.75 ± 0.07	24.50 ± 0.05	24.56 ± 0.44	24.73 ± 0.37
Q6AZA2	ndufv1		14	14	156	*	*	*	0.5	0.6	0.6	28.20 ± 0.12	27.08 ± 0.47	27.46 ± 0.27	27.42 ± 0.25
Q6PBX8	ndufv2		5	5	57	*	*	*	0.5	0.6	0.6	26.77 ± 0.21	25.67 ± 0.51	26.02 ± 0.17	26.1 ± 0.24
Q8AW03	ndufa6		5	5	65	**	**	*	0.6	0.5	0.6	26.51 ± 0.12	25.68 ± 0.01	25.62 ± 0.21	25.75 ± 0.24
F1QHE9	ndufs7		4	4	37	*	**	*	0.6	0.5	0.6	26.01 ± 0.13	25.32 ± 0.14	25.05 ± 0.22	25.29 ± 0.12
Q3B7G1	ndufb5		6	6	43	*	**	*	0.5	0.5	0.5	26.18 ± 0.23	25.12 ± 0.11	25.16 ± 0.13	25.22 ± 0.25
A0A0U2NDI4	ND1		3	3	20	**	*	**	0.6	0.7	0.6	26.00 ± 0.09	25.22 ± 0.10	25.39 ± 0.19	25.34 ± 0.1
**Glycolysis Pathway**															
Q9PVK5	ldha	L-lactate dehydrogenase	16	14	185	*	**	*	1.6	1.4	1.2	30.76 ± 0.10	31.43 ± 0.29	31.22 ± 0.06	31.05 ± 0.05
**Stress and Inflammatory Response**															
F6NYT7	gpx1a	Glutathione peroxidase	11	11	83	**	**	*	115	51	47	21.65 ± 0.11	28.50 ± 0.70	27.32 ± 0.54	27.21 ± 1.29
Q9DDU5	gstp1	Glutathione S-transferase, Pi	13	13	175	*	**	**	1.9	1.9	1.8	31.70 ± 0.14	32.58 ± 0.32	32.59 ± 0.14	32.52 ± 0.07
A0A2R8RU89	cst14b.2	Cystatin 14b	3	3	23	n.d.	n.d.	n.d.	n.d.	n.d.	n.d.	NaN	24.37 ± 1.19	23.82 ± 1.16	23.75 ± 1.71
Q5PR64	hspb1	Heat shock 27 kDa protein	9	9	86	**	**	**	0.3	0.4	0.4	27.93 ± 0.21	26.33 ± 0.16	26.66 ± 0.14	26.42 ± 0.17
**Lipid Metabolism**															
Q5IHX6	ptges3a	Cytosolic prostaglandin E synthase	5	4	48	***	***	***	4.4	4.0	3.6	24.01 ± 0.14	26.14 ± 0.31	26.01 ± 0.13	25.76 ± 0.23
F1QVT8	enpp6	Choline-specific glycerophosphodiester phosphodiesterase	8	8	49	*	*	**	1.8	1.5	1.6	24.97 ± 0.18	25.83 ± 0.39	25.53 ± 0.12	25.65 ± 0.11

LFQ, label-free quantitation; *p*-values when compared to wild type, * <0.05, ** <0.01 and *** < 0.001. n.d, not determined. NaN: Non Assigned Number (not detected).

**Table 2:** Proteins regulated in DJ-1 knockout retina in which expression levels are restored by reinsertion of wild-type DJ-1 in Müller cells.

						*p*-Value			Max Fold			Average LFQ (log2)			
Protein ID	Gene Name	Protein Name	Total pep.	Unique pep.	Score	WT vs. KO	KO vs.M_DJ-1	DJ-1 vs. DJ-1c106a	KO/WT	M_DJ1/ WT	M_DJ-1c106/WT	WT	KO	M_DJ-1	M_DJ-1c106a
F1R6R2	apcs	Amyloid P component, serum	9	9	171	*	*	n.s.	2.6	0.6	1.2	28.87 ± 0.07	30.27 ± 0.61	28.14 ± 0.40	29.17 ± 0.72
Q8UVZ4	psap	Prosaposin	6	6	64	*	*	n.s.	1.5	1.1	1.4	27.27 ± 0.11	27.88 ± 0.17	27.41 ± 0.16	27.73 ± 0.35
A4FUN8	rhol	Rhodopsin-like	4	4	49	*	*	n.s.	0.5	0.8	0.5	26.63 ± 0.36	25.61 ± 0.25	26.40 ± 0.24	25.73 ± 0.24
Q6PBA5	cnn2	Calponin	4	3	30								25.02 ± 0.74		23.90 ± 0.54
A0A2R8QT38	rpl36a	60S ribosomal protein L36a	2	2	14								24.47 ± 0.05		24.74 ± 0.26
E7F0E8	xpo1a	Exportin 1	21	4	29							24.02 ± 0.43		23.93 ± 0.09	
E7F3I2	gpr37a	G protein-coupled receptor 37a	2	2	15							24.40 ± 0.02		24.48 ± 0.18	
Q5RKQ2	mtx1b	Metaxin	2	2	11							22.31 ± 0.24		22.10 ± 0.15	
A0A0R4 IDW9	sec23ip	SEC23-interacting protein	3	2	24							23.38 ± 0.34		22.85 ± 0.04	

LFQ, label-free quantitation; * *p* < 0.05. n.s.: not significant.

**Table 3:** Proteins only regulated in DJ-1 knockout retina.

						*p*-Value (vs. KO)			Max Fold			Average LFQ Values (log2)			
Protein ID	Gene Name	Protein Name	Total Pep.	Unique Pep.	Score	WT	M_DJ-1	M_DJ-1c106a	KO/WT	M_DJ-1/WT	M_DJ-1c106a /WT	WT	KO	M_DJ-1	M_DJ-1c106a
P17561	epd	Ependymin	7	7	280	*	**	*	6.7	0.5	0.9	29.05 ± 0.37	31.80 ± 0.67	28.06 ± 0.23	28.97 ± 0.91
A3KPR3	histh1l	Histone-H1-like	6	5	38	*	*	***	2.3	1.3	1.1	25.43 ± 0.18	26.66 ± 0.17	25.80 ± 0.19	25.52 ± 0.04
Q8JH28	ctsd	Cathepsin D	11	11	107	*	*	*	1.8	1.4	1.4	29.25 ± 0.08	30.13 ± 0.17	29.70 ± 0.06	29.69 ± 0.09
Q502B7	mcee	Methylmalonyl CoA epim.	4	4	30	*	*	*	1.4	1.2	1.2	25.55 ± 0.05	26.00 ± 0.06	25.78 ± 0.08	25.77 ± 0.08
Q5XTN6	crygm1	Crystallin,γM1	7	5	59				2.0			23.44	24.42 ± 1.68	n.d.	n.d.
Q4ZHG3	crygm2a	Crystallin,γM2a	4	3	39				1.7			23.42	24.21 ± 1.59	n.d.	n.d.
Q6DGJ1	grifin	Grifin	4	4	34				1.9			24.71	25.60 ± 0.87	23.87	n.d.

LFQ, label-free quantitation: * *p* < 0.05, ** *p* < 0.01 and *** *p* < 0.001. n.d.: not detected.

## Data Availability

Data is contained within the article or supplementary material.

## Supplementary Materials

The following are available online at https://www.mdpi.com/article/10.3390/antiox10121862/s1, Figure S1: Age-dependent retinal degeneration in DJ-1 knockout retina, Figure S2: In situ hybridization of Glutathione S-transferase mRNA in the retina, Table S1: Expression of Retinal cell markers, Table S2: List of the 1868 identified protein used for quantitative analysis.
