# Supplementary material for: Reintroduction of DJ-1 in Müller Cells Inhibits Retinal Degeneration in the DJ-1 Deficient Retina
Source: Antioxidants (Basel). 2021 Nov 23;10(12):1862. doi: 10.3390/antiox10121862 (PMC8698414; doi:10.3390/antiox10121862)
Supplement: Supplementary file 1 [file antioxidants-10-01862-s001.zip › antioxidants-1454986-supplementary.pdf]

Suppl. Table S1 Expression of Retinal cell markers

Retinal Cell Type	Protein ID	Protein	Gene	Total pep.	Unique pep.	Seq.coverage	Average LFQ values (log2) WT	KO	M_DJ-1	M_DJ-1c106a
Retinal Ganglion Cell	P873660	Gefiltin	inab	30	18	181	28.80±0.78	27.15±0.17*	28.76±1.35	27.40±3.16
Retinal Pigment Epithelium	Q6PBW5	Retinoid isomerohydrolase	rpe65a	35	35	74	32.94±0.32	32.62±0.36	32.60±0.22	32.30±0.08
	Q5TZG5	Ezrin	ezrb	15	10	20	25.35±0.17	25.11±0.09	25.26±0.14	25.18±0.10
Müller cell	B2GP50	Glial fibrillary acidic protein	gfap	18	15	43	27.64±0.21	25.98±1.84	27.36±0.38	26.81±0.70
	Q7ZVF2	Glutamine synthetase	glulb	14	8	38	29.81±0.26	29.48±0.16	30.03±0.26	29.84±0.15
Rod Photoreceptor	Q8AV67	Rhodopsin	rho	8	6	28	33.25±0.15	33.08±0.55	33.47±0.54	32.41±0.89
	A4FUN8	Rhodopsin	rhol	4	4	17	26.63±0.44	25.61±0.31*	26.40±0.30	25.73±0.30*
Cone Photoreceptor	Q801U8	Green-sensitive opsin-2	opn1mw1	9	3	32	30.77±0.42	30.81±0.32	31.04±0.28	30.81±0.07
Microglia and macrophages	Q7SXE0	Macrophage-expressed gene 1 protein	mpeg				n.d.	n.d.	n.d	n.d.

**p<0.05 as compared to WT*

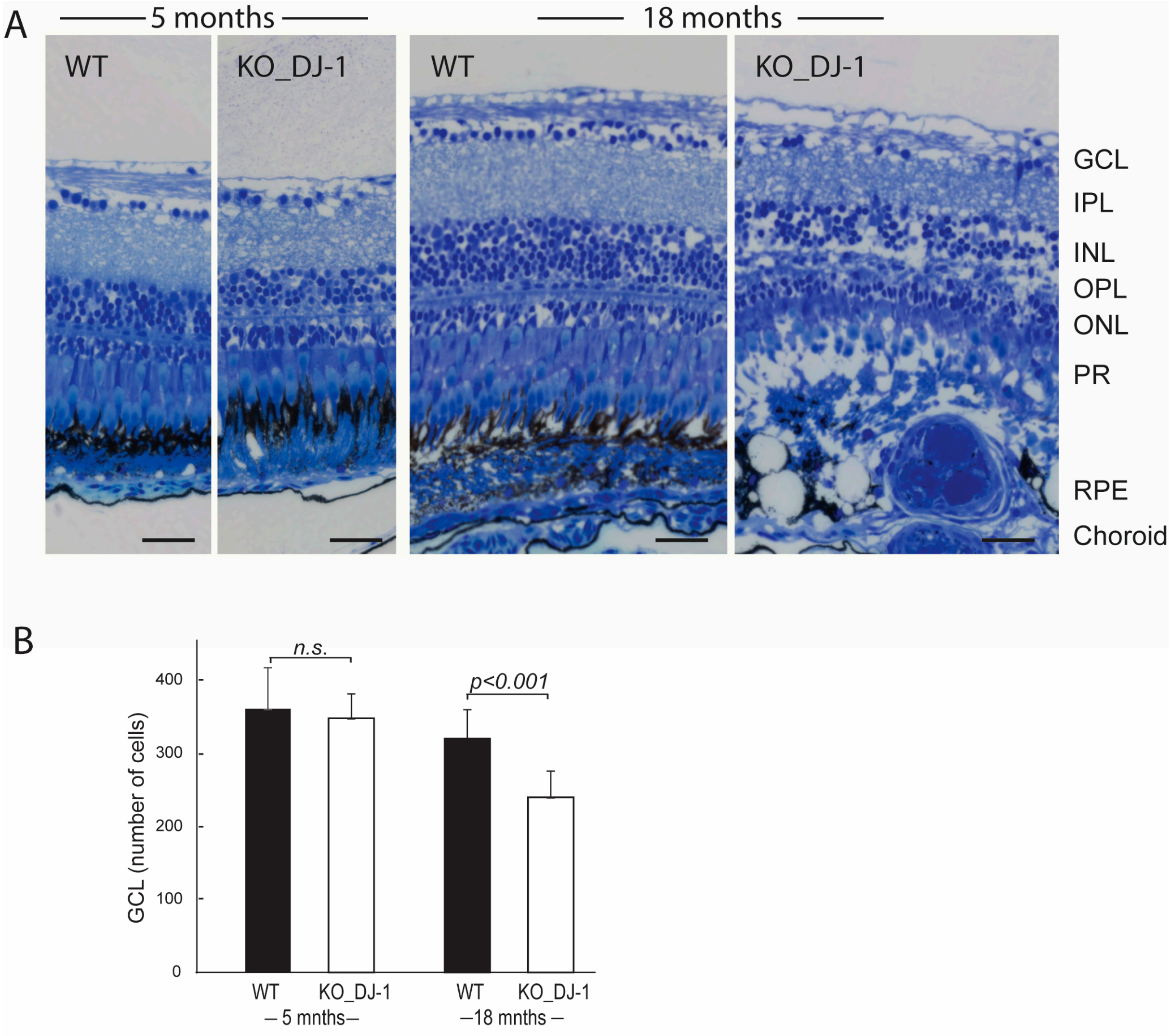

Suppl. Figure S1 Age-dependent retinal degeneration in DJ-1 knockout retina (A) Light microscopic images of toluidine blue stained retinal cross sections from five and 18 months old wild type and DJ-1 knockout adult zebrafish. Bar 20 µm. GCL: ganglion cell layer, IPL: inner

plexiform layer, INL: inner nuclear layer, OPL: outer nuclear layer, PR: photoreceptors, RPE: retinal pigment epithelium. **(B)** Number of cells in ganglion cell layer. $P < 0.001$ Student t-test ($n = 6$). n.s.: not significant.

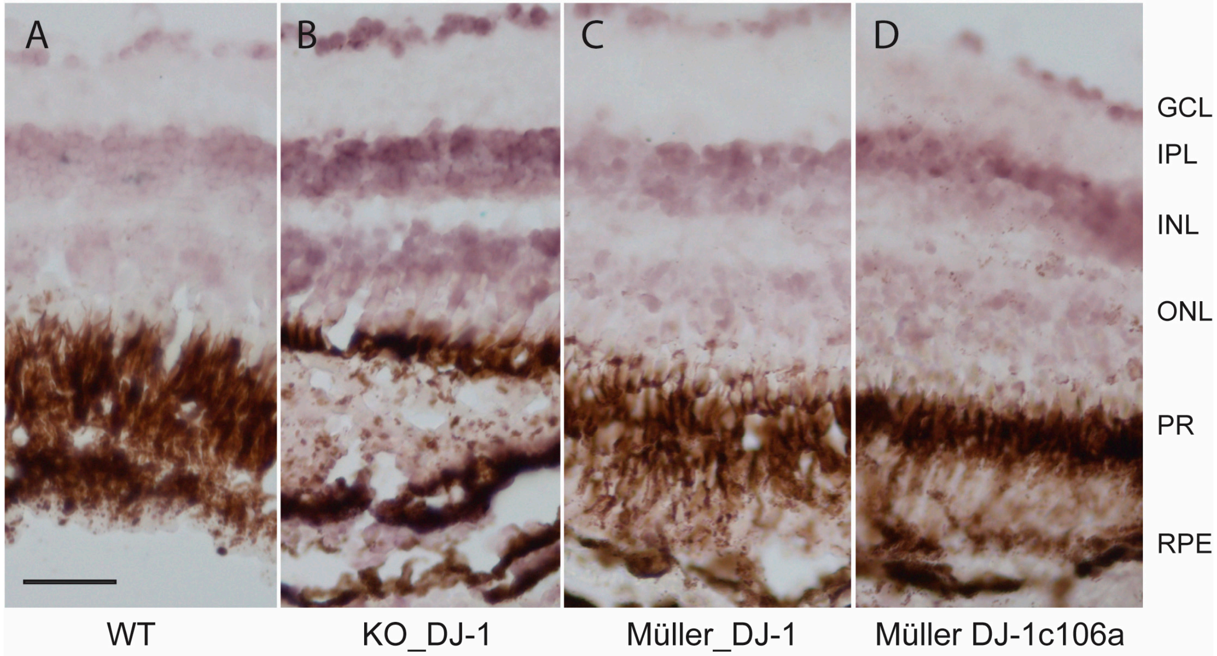

Suppl. Figure S2 *In situ* hybridization of Glutathione S-transferase mRNA in the retina (A) Wild type. (B) DJ-1 knockout. (C) Müller cell expressed wild type DJ-1. (D) Müller cell expressed DJ-1c106a mutant. Bar 20µm applies to all panels. GCL: ganglion cell layer, IPL: inner plexiform layer, INL: inner nuclear layer, OPL: outer nuclear layer, PR: photoreceptors, RPE: retinal pigment epithelium.
